# 871. Sexual Health Service Experience and Preferences for Non-Traditional Service Delivery Among a Predominately Immigrant Latino MSM Population in Miami

**DOI:** 10.1093/ofid/ofab466.1066

**Published:** 2021-12-04

**Authors:** Samantha Herbert, Katie Klose, Liz Rivera, Brahian Erazo, Brian Baez Leon, Katherine King, Blonsky Batalien, Max Olivier-Bros, Susanne Doblecki-Lewis

**Affiliations:** 1 University of Miami Miller School of Medicine, Miami, FL; 2 University of Miami, Miami, FL

## Abstract

**Background:**

Miami-Dade County (MDC) has the highest rate of new HIV diagnoses in the United States (US), with highest incidence among Black and Latino men who have sex with men (MSM). Immigrants may be especially vulnerable to HIV acquisition and may lack or avoid accessible sexual health services. The University of Miami Mobile PrEP (MP) Clinic provides sexual health services including STI and HIV testing as well as PrEP initiation and follow-up in four highly impacted areas of MDC. The majority of MP clients are immigrant Latino MSM. We evaluated sexual healthcare access, preferences, and facilitators or barriers to receiving sexual health services through non-traditional platforms.

**Methods:**

A brief survey was offered to clients at four MP locations from September 2020 to June 2021. Multiple-choice questions addressed healthcare access, usage, and experience as well as preferences for service receipt including home-based, mobile clinic, and telehealth options. Brief qualitative short answer responses were also elicited. Results were tabulated and presented descriptively.

**Results:**

A total of 115 clients were surveyed. Mean age was 36; 82.6% identified as male. Most respondents were either White/Caucasian (56.5%) or Black/African-American (19.1%) and 78 (67.8%) identified as Hispanic/Latinx. Of the 66% that reported being born outside the US, 34.2% had immigrated in the past 5 years. Only 41.7% of respondents had a primary care provider. Before coming to the MP clinic, 27% had not been seen for sexual health services in over 2 years. Most clients indicated satisfaction with MP services. The most important characteristics for a care site identified included comfort with staff, location, and affordability. 43.5% preferred a clinic time outside of 9am-5pm. Only 13% of clients preferred home-based labs using a self-collection kit with a majority preference for in-person follow-up at the MP clinic.

**Conclusion:**

Key populations at risk for HIV infection including immigrants and Black and Latino MSM may experience barriers to traditional clinic care. Clients expressed satisfaction with MP services, and a preference for clinic-collected rather than self-collected specimens. Further research to tailor service delivery to client preferences is needed.

**Disclosures:**

**All Authors**: No reported disclosures

